# Cultural Confidence on “Art & Engineering” Construction of Product Design under “New Liberal Arts”

**DOI:** 10.1155/2022/6101368

**Published:** 2022-05-09

**Authors:** Lijun Xu, Jun Gao, Lu Chen, Guodong Liang, Hailong Feng

**Affiliations:** ^1^Institute of Art and Design, Nanjing Institute of Technology, Nanjing, Jiangsu 211167, China; ^2^ABB Beijing Drive System Co., Ltd., Beijing, China

## Abstract

A master's degree program in “new liberal arts” is a great opportunity to strengthen cooperation between art colleges and universities, to promote the construction of product design and digital molding majors, and to promote teaching and research on the “integration” of the theoretical foundations of engineering intelligent manufacturing. To take advantage of cooperative universities' advantageous disciplines and professional platforms, we should construct new art and emerging interdisciplinary majors, as well as promote the construction and exploration of joint training of doctors and masters in relevant disciplines in the interdisciplinary direction of art and engineering. The foregoing approaches are intended to create a new “art and engineering” model for the establishment of a product design speciality at our university. In order to meet the requirements of “Art & Engineering” advocated by the national new engineering construction, art disciplines have their own inherent rules and characteristics. We should actively create disciplinary and professional characteristics, help universities cooperate with each other in high-quality development, and share and win together, in order to continuously achieve new academic results.

## 1. Introduction

China's “Six Excellences and One Top” Plan 2.0 was launched in Tianjin on April 29, 2019, with the goal of boosting universities' capacity to serve economic and social development by promoting the construction of new engineering, new medicine, new agriculture, and new liberal arts. When it comes to the growth of higher education, “new liberal arts” has become a subject that needs to be taken seriously [1].

It is necessary to grasp the new requirements for the development of philosophy and social sciences in order to strengthen the construction of new liberal arts. It is also necessary to cultivate new cultures with Chinese characteristics, such as Chinese style and Chinese style in the new era, as well as philosophical and social scientists. It is also necessary to establish a Chinese school of philosophy and social sciences in order to strengthen the construction of new liberal arts. The construction of “new liberal arts” has as its primary goal the adaptation to the new requirements of the development of philosophy and social science in the new era as well as the promotion of cross-fertilization between philosophy and social science with the new round of scientific and technological revolution and industrial change. As a result, the “new liberal arts” have emerged as a topic that must be properly studied and explored in the development of higher education in the contemporary day. For the “new liberal arts,” efforts should be made to cross disciplines, form and expand new fields of knowledge at the boundaries between disciplines, particularly in conjunction with science and technology, and realize the intersection of arts and science. This, for the liberal arts, entails enhancing the scientificity of liberal arts and thus promoting the construction of “new liberal arts.”

It was Bauhaus principal Walter Gropius who introduced the educational concept of “Art & Engineering” product design for the first time. Gropius insisted on the new unification of art and technology, which resulted in the formation of a new educational system that combined art education with handicraft. With its approach of merging “pure art“ with “useful technology” [2, 3], the Bauhaus provided inspiration for the future “Art & Engineering” teaching model.

The term “engineering” in the “Art & Engineering” mainly refers to engineering in science, which is a discipline in which people study and apply theories and knowledge of natural science, sociology, economics, logic, and other disciplines to innovate or improve the design and use of solutions in various fields [4]. It also includes the meaning of “engineering technology,” in which people use engineering technology to make practical objects or tools to extend or compensate for human capabilities and change people's way of life, with the intention of being practical.

Product design is a kind of industry that is about innovation, and without innovation, there is no vitality. The educational concept of product design should also include the concept of innovation. Since the Bauhaus pioneered the innovative educational concept and mode of “Art & Engineering,” institutions across the world are following suit [5]. However, in this new era, if we adhere to this concept and follow the trend without seeking innovation, the design talents we cultivate will be out of step with the society. Based on the diversified, informative, and intelligent conditions of the times, the educational concept of “Art & Engineering” in product design must keep pace with the times and make the concept of design education have new thinking characteristics.

Rather than just rehashing the old buzzwords of “intersection of disciplines” or “intersection of arts and sciences,” the concept of the new liberal arts should be understood from the perspective of China's intrinsic requirements for postsecondary education [6]. The investigation and practice of “Art & Engineering” of product design in the context of “New Liberal Arts” are still in the early stages of its development and implementation.

Problems

1. The engineering technology category of product design majors does not have sufficient teaching qualifications, and it is difficult to connect the more artistic design majors with the more technical engineering technology at this time. Product design majors will concentrate on the design of rail transportation class and intelligent product class, which will be based on the application of big data, cloud computing, and artificial intelligence technology, allowing the design to be more scientific, rational, and extensive in the context of the “New Liberal Arts.” As a result of this rapidly changing development environment, the knowledge structure, cognitive ability, and thinking mode requirements of designers will undergo significant shifts in the near future. As a result, design education will need to adapt quickly in order to meet the changing needs of society.
2. There is a difference between the training for talents and the education of design as well as the most recent technology available on the market. (2) Product design teachers must convert their art backgrounds and engineering teaching concepts into product design teaching concepts. The talents cultivated by design education will be unable to meet the needs of social development and will eventually be eliminated if design education does not adapt to the changing times in order to adjust the goal of talent training and does not use engineering technology to complement the design practice. It will also be difficult to implement the employment of design education students. In order to better support product design teaching, it is urgently necessary to investigate how “Art & Engineering” can promote the reform of product design education from a systematic and holistic perspective as well as to clarify the ideas for “Art & Engineering” to better support product design education and cultivate more professional product design applied talents.

Solutions

1. “Art & Engineering” should reflect the personalization and diversity of interdisciplinary
2. To further strengthen the construction of product design discipline, give full play to disciplinary strengths, and concentrate disciplinary characteristics; to strengthen interdisciplinary crossover and integration with art colleges and engineering majors and take the construction of a special master's degree in art as an opportunity to build the disciplinary characteristics of “Art & Engineering”; to do a good job of training product design talents with high quality and establish an application-oriented education training system that serves the needs of economic and social development.
3. “Art & Engineering” should pay attention to talent training
4. To further boost the creation of product design majors and the training of future product design talent. Taking the construction of new engineering and new liberal arts as a model, establish and improve the dynamic adjustment mechanism of majors, optimize the structure and connotation of majors, expand the enrollment scale of majors, and establish new majors in art; concentrating on the cross-fertilization of art and engineering to form the cultivation characteristics of art and design talents, promote the in-depth reform of project-based teaching, and strengthening the internationalization of art and design talents; focusing on the cross-fertilization of it is necessary to continue to increase the construction of teaching staff at the “Art and Engineering” school. It is also important to develop a high-level professional teaching and research team with a worldwide perspective through training, further training, and study visits, in addition to expanding the channels for the entry of high-level talents into the product design industry.
5. Research on the new platform of discipline construction in College of Arts and Design
6. To adapt to the requirements of “Art & Engineering” advocated by the national new engineering construction, art disciplines have their own inherent rules and characteristics, should actively create disciplinary and professional characteristics, and help universities cooperate in high-quality development to help each other and share and win together, in order to continuously achieve new academic results.

At this stage of the school's development ideas and goals, the status of the School of Art and Design is increasingly important, directly related to the comprehensive and coordinated development of the school's related disciplines, the important indicators of the school's name change, and the core content of the school's future sustainable development. Therefore, the great development of art and design disciplines should fully rely on the school's traditional advantageous disciplines, build a new platform for discipline construction of the College of Art and Design, and strive to form a number of characteristic high-level research results around the direction of discipline construction of the College, with the common ideal of “building a first-class art and design college of the same kind with domestic influence and provincial status” [7, 8], form the mainstream value orientation common to all the faculty and students, and concentrate our efforts to create a college culture atmosphere that emphasizes discipline and professional development, academic research and artistic creation, harmonious construction and quality improvement, and continuous deepening, expansion, and ascension.

## 2. New Thinking and New Forms of Cultural Confidence Research of “Art & Engineering”

**Figure 1:**
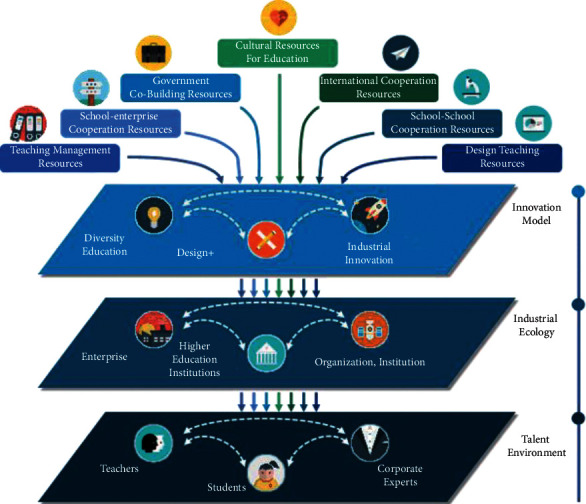
New thinking and new forms of education in “Art & Engineering” cultural confidence theory research.

According to the “new liberal arts” theory and practice, the Jiangsu brand-product design major establishes a new pattern of “Art & Engineering” in accordance with the investigation and practice of the development of national first-class products. It will be developed in conjunction with the master's degree program at the College of Art and Design in order to strengthen cooperation between art colleges and universities, cooperation with engineering practice majors, and practical teaching of intelligent design and will strive to promote a close connection between knowledge transfer and production practice. It will also be developed in conjunction with the master's degree program at the College of Art and Design in order to promote “integration” of theory and practice in the field of product design and digital molding. It is necessary to establish a design research and development platform with innovation as the goal, a practical platform with engineering training as the means, and a social crowdfunding or school-enterprise cooperation platform with market demand as the guide in order to explore and practice “Art & Engineering” in product design education, resulting in the formation of a new way of thinking about and practicing “Art & Engineering” in product design education. It is shown in Figure 1.

As part of this effort, the college will make better use of the advantageous disciplinary and professional platforms of the partner institutions to carry out the construction of new art and emerging cross-disciplinary disciplines and professions. It will also work to promote the construction and exploration of joint training programs for PhDs, Master of Arts, and Master of Engineering degrees in relevant disciplinary fields as well as joint training programs in engineering-related cross-disciplinary directions. A number of initiatives will be undertaken by the two sides to benefit from their respective disciplines, such as the development of new art and emerging interdisciplinary disciplines and majors, the establishment and sharing of internship and practical training teaching practice bases, the cultivation of PhDs and Master of Art and Engineering-related interdisciplinary directions within relevant disciplinary fields, as well as the collaboration in the development of an innovative and entrepreneurial education system. The partnership will be translated into practical measures to support the development of the school in a timely manner, as well as to promote the quick expansion of the school's business, and work toward the establishment of a new pattern of “Art & Engineering.”

### 2.1. Research on the Construction of Cultural Confidence Majors with “Art & Engineering”

1. Research on new thinking and new forms of “Art & Engineering” in the era of “New Liberal Arts”
2. Research on teacher training for “Art & Engineering” talents in the field of design in universities and research on leading the cultivation of product design talents
3. Research on the construction of product design talent training objectives and curriculum system with “Art & Engineering”

### 2.2. Investigation into the “Art & Engineering” Teaching Mode and the Use of Abilities in Intelligent Design

1. To investigate the current state of product design higher education in both China and abroad, as well as the characteristics and applications of intelligent technology, in order to predict the future development trend of intelligent product design education and to establish a fundamental theoretical system for intelligent product design education in China and abroad
2. To redefine the training objectives of product design talents in the new liberal arts era, improve the talent training program, and establish the core curriculum system of “Art & Engineering”
3. To study the changes in the teaching resource environment, teaching and learning methods, teaching management and evaluation methods, personalized teaching mode, and teacher training in the “Art & Engineering,” to propose the reform direction of product design education, and then to build the education system of product design in the era of “New Liberal Arts”

### 2.3. Research on the Construction of a Teaching and Research Platform for Product Design and Digital Molding of “Art & Engineering”

1. Research on courses that are closely linked to the “Art & Engineering”: Human-Computer Interaction Design Research at master's level, Advanced Manufacturing Technology and Artistic Design, Thematic Studies in Electromechanical Product Design, Product and Interaction Design Project Practice, Design competitions and exhibitions.
2. Research on computer-aided design series courses, product design series core courses, model making, thematic design series courses, CG production of animation characters, cultural and creative design of animation, model making of craft products, jewelry design, environmental model making, and many other professional basic courses and professional courses in the undergraduate stage of “Art & Engineering”.
3. Researchers at home and abroad are investigating intelligent educational platforms for “Art & Engineering” in a variety of contexts, including the platform characteristics and operation system of educational robots and platforms, teaching software and teaching tools, intelligent virtual assistants, among other things; they are also investigating the teaching and research platform of intelligent product design and digital molding.

## 3. Diversification of “Art & Engineering” Practice Research with Cultural Confidence

When placed in the context of the “new liberal arts,” product design majors' educational thinking must seek innovation and keep up with the times; as a result, the educational mode of “Art & Engineering” tends to be market-oriented, internationalized, personalized, and manifesting the characteristics of regionalization [9–11].

### 3.1. Enhancing Students' Interest and Skills in “Art and Engineering” Intellectual Creation

Students' real-world experience with a variety of material processing techniques and skills is enhanced through the use of the basic molding system, which increases the means of expression and the ability of intelligent product design for art and design students, which is conducive to activating students' creative thinking, encouraging students to carefully observe the surrounding living environment and the current social situation, and combining objective natural laws and their own unique thin-film technology. The measures outlined above can assist us in cultivating a diverse pool of high-quality applied talents to satisfy the needs of economic and social development.

### 3.2. Improving the Quality of Talent Cultivation of “Art & Engineering” and Sharing Teaching Resources with Cultural Confidence

Students can conduct experiments at any time and from any location because of the development of this project, which overcomes the limitations of time and space. At the same time, the software has a variety of learning functions that allow pupils to practice over and over again. “Observation, recording, and combination-making exercises” [12] of virtual experimental items in a variety of virtual experimental contexts have helped students to improve their design skills. When this initiative is put into practice, it significantly improves the scientific approach to learning as well as the professionalization of students' practical skills. Using the Internet, participants can easily access and use large-scale class experiments, and the project is open to the general public as well as educators. As of now, not only do the virtual simulation teaching materials serve the staff and students at this college but they also assist in the virtual simulation experimental teaching of other related majors.

### 3.3. “Art & Engineering” Should Be Personalized and Diversified Teaching

When it comes to improving students' design ability, the design education process is critical because it cultivates strong hands-on practical skills while also growing high theoretical literacy and the capacity to make full use of regional cultural resources to enrich their design concepts. In addition, professional craftsmen who are engaged in design and production work in the field of traditional crafts should be hired to teach at the institution. At the same time, in order to realize the purpose of assisting social production, the school should develop an industry-university-research platform in collaboration with businesses. A design that has not been translated into a product and the value of that design has not been realized are impossible if pupils do not grasp the manufacturing process. Students from all directions study professional basic courses together in the freshman year, such as design aesthetics, design composition, design expression techniques, the history of world arts and crafts, the history of Chinese arts and crafts, and other basic courses [13–15]; in the sophomore year, we implement discipline subdivision and choose discipline direction, such as curriculum of ceramics, wood carving, leather design, furniture design, and other disciplines [16–18].

### 3.4. “Art & Engineering” Should Implement Industrialization

**Figure 2:**
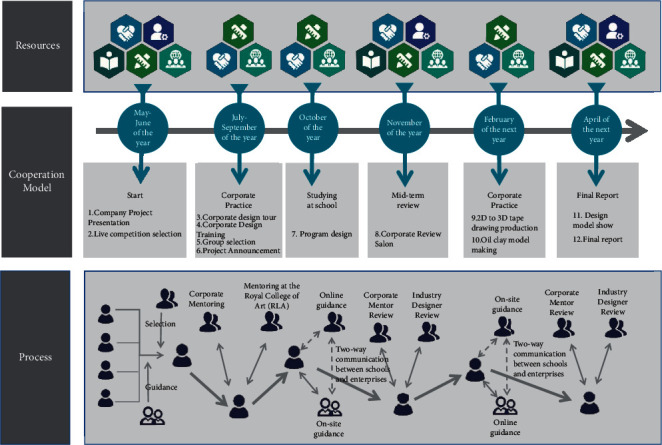
The “Art & Engineering” school-industry partnership project follows a logical progression.

The industrial transformation of design works, as well as the capacity to follow up in the process of product manufacture, processing, and sales, is an important element of the product design profession and one that can drive students to pursue a degree in product design which includes: (1) establishing craft design factories or studios on campus, as well as crowdfunding platforms on various commercial websites to publicize the works and raise funds for the processing and production of design works; (2) establishing school-enterprise partnerships with enterprises, in which the school commissions enterprises to process design works and provide them with marketing, as depicted in Figure 2; and (3) establishing school-enterprise partnerships with enterprises, as depicted in Figure 2.

### 3.5. “Art & Engineering” Should Be Internationalized

Design, like art, has no geographical boundaries. It is also necessary to integrate and cross borders in the “Art and Engineering” approach to product design education in order to better portray the spirit of product design education. Internationalization of the “Art and Engineering” approach to product design education is recommended in order to establish a broader and more diverse sector of design education [16–18]. The process of “Art & Engineering” product design education, such as the teaching of ceramics courses, must combine ceramic engineering and art, and it is necessary to cultivate advanced composite applied talents who understand both ceramic engineering and have excellent artistic design ability in order to meet the needs of China's ceramic industry development. Studying abroad, getting to know oneself and one's adversary, and learning the essence are all essential for standing out in design education.

## 4. Innovation and Prospect of “Design+” Multidisciplinary Cross-Fertilization with Cultural Confidence

In the context of the “New Liberal Arts,” the educational thinking of product design majors must be innovative and keep up with the times so that the educational mode tends to be industrialized, internationalized, and personalized in the market, as well as manifesting the characteristics of regionalization, as shown in the following diagram. It is necessary to establish a design research and development platform with innovation as the goal, a practical platform with engineering training as the means, and a social crowdfunding or school-enterprise cooperation platform with market demand as the guide in order to explore and practice “Art and Engineering” in product design education, resulting in the formation of a new way of thinking about and practicing “Art and Engineering” in product design education. The following are examples of specific innovations.

### 4.1. Research on the Innovative System of Cultivating Intelligent Applied Talents in Product Design under the “Art & Engineering”

The product design major, which is a combination of art and engineering in the context of “New Liberal Arts,” should adhere to the principles of moral education, establish cultural self-confidence, support the high-quality development of the regional economy, and cultivate innovative and applied product design professionals who can comprehensively develop “moral, intellectual, physical, aesthetic, and labor” and grasp the elements of product function, form, material, structure, and color in the context of “New Liberal Arts.”

1. The practice of “Art & Engineering” product design and professional construction in the context of “New Liberal Arts” is being explored and researched.
2. It is necessary to emphasize the core purpose of “Art & Engineering” in design majors' curriculum and teaching system and introduce this learning concept to students early in the teaching process [19] so that students realize that they are product designers with comprehensive professional qualities rather than conceptual designers who can only draw concept drawings. We use the case analysis method to examine the current situation of product design education at home and abroad, as well as the nature, connotation, and construction ideas of the “New Liberal Arts.” We also investigate the development trend of “Art & Engineering” in product design education, and we establish the fundamental theoretical system of product design education under the umbrella term of “New Liberal Arts.”
3. “Art & Engineering” is a teaching style that focuses on the application of intelligent design applied talents
4. When faced with the challenges of diversity, information technology, and intelligence, the education concept of “Art & Engineering” for product design majors must evolve to keep up with the times so that the design education concept possesses new thinking characteristics as well. By adopting a new model of product designers' “Art & Engineering” skills, we can reevaluate the training objectives of product design talents, improve the training program, and establish a core teaching model that meets the innovative consciousness and creative ability of “Art & Engineering” skills in the context of “New Liberal Arts.”

### 4.2. Construction of Industry-Education-Research Platform for Product Design under the “Art & Engineering”

As required by the discipline's talent cultivation goal, and in order to satisfy the needs of modern design and manufacturing firms, the College of Art and Design is collaborating with the College of Design to declare a master's degree in the field of art and design. A new teaching and research platform for product design and digital molding will be established in order to promote the “integration” of theory and practice in smart manufacturing. This will help to improve the quality of practical instruction while also promoting the close connection between knowledge transfer and manufacturing practice. The development of digital molding technology has altered the rigid standard of traditional manufacturing, allowing for greater design flexibility as the technology advances. In addition, the development of digital molding technology has increased the design dimension while simultaneously enriching the design means and presentation level. It encourages the development of teachers' scientific research abilities while also providing substantial technological and physical assistance for the collection of scientific research results in the classroom.

Innovative research on the construction of a teaching and research platform for product design and digital molding of the “Art and Engineering”. An important feature of the product design profession and an important aspect that can motivate students to learn is the industrial transformation of design works and the ability to follow up during the production, processing, and sales of products. This teaching and research platform is composed of basic molding system, digital molding system, and reverse design system and is centered on three major master's specialties: product and interaction design, environment and landscape design, and visual and information design, to purchase relevant research instruments and equipment, create a research platform, support relevant basic theory and key technology research, promote the development of relevant disciplines in the university, and help local industries transform and upgrade.

### 4.3. Establishing a Design Education System of “Vision Sharing-Resource Sharing-Platform Building-Effectiveness Co-creation” as a Linked Evolutionary Mechanism

**Figure 3:**
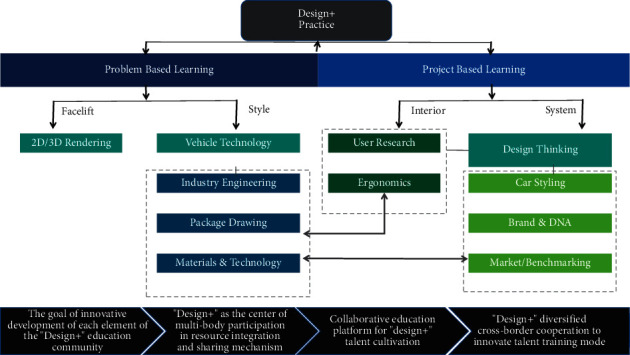
Reform and practice plan of design interdisciplinary graduate education.

As a result of technological advancement, social transformation, and international rivalry, new needs for the growth of design education have been established. Specifically, we investigate the objectives, processes, methods, and resources of professional talent training for design graduate students. We also examine the elements and their mutual relationships among them; categorize the elements according to the logical relationship between the domain layer, platform layer, and subject layers [20, 21]; construct an interdisciplinary education system for design majors; follow the basic ideas of interdisciplinary professional construction; form the key tasks of profession; and make the interaction between the many levels of the system and the talent, industry, and innovation chains more comprehensible and understandable. A linking evolution mechanism of “vision sharing-resource sharing-platform building-effectiveness co-creation” is proposed to carry out professional education reform and practice, based on our findings. The reform and practice plan can be seen in Figure 3.

## 5. Conclusion

As our school's development ideas and goals progress, the status of our design majors has become increasingly important, as it is directly related to the comprehensive and coordinated development of the school's related disciplines, to the important indicators of the school's name change, and to the heart of the school's long-term sustainable growth. The great development of design interdisciplinary disciplines should, as a result, fully rely on the school's traditional advantageous disciplines, create a new platform for the construction of interdisciplinary disciplines in the college, and strive to form several characteristic high-level research results centered on the college's discipline construction direction. The common ideal of “constructing a first-class college of art and design with domestic influence and provincial status” should serve as a guide for all faculty and students, and we should concentrate our efforts on developing a college culture that emphasizes discipline and professional development, academic research and artistic creation, harmonious construction and quality improvement, and continuous deepening, expansion, and ascent. In addition, we will further explore how to combine the new teaching mode system of artificial intelligence in the near future.

## Data Availability

The data set used to support the findings of this study is available from the corresponding author upon request.

## References

[1] Su P.-H. (2017). Case studies of applying electronic flexible material and technology to create the new media arts. *Multimedia Tools and Applications*.

[2] Schoormann T., Stadtländer M., Knackstedt R. (2021). Designing business model development tools for sustainability—a design science study. *Electronic Markets*.

[3] Filippo C., Paola B., Gualtiero F. (2021). Data science for engineering design: state of the art and future directions. *Computers in Industry*.

[4] Marko L., Suvi N. (2021). Design science and Co-designing of hybrid workplaces. *Buildings*.

[5] Grace P., Lewis D., Pollack T. (2021). Employing user-centered design and learning science theory to enhance remote delivery of diabetes education and survival skills at hospital discharge. *Journal of the Endocrine Society*.

[6] Guo L. (2016). A system design method for cloud manufacturing application system. *International Journal of Advanced Manufacturing Technology*.

[7] Meng D., Yan L., He C., Guo J., Lv Z., Wu P. (2021). Multidisciplinary design for structural integrity using a collaborative optimization method based on adaptive surrogate modelling. *Materials & Design*.

[8] Chen H., Li W., Cui W., Yang P., Chen L. (2021). Multi-objective multidisciplinary design optimization of a robotic fish system. *Journal of Marine Science and Engineering*.

[9] Wang S., Yang M., Niu W. (2021). Multidisciplinary design optimization of underwater glider for improving endurance,” Structural and Multidisciplinary Optimization. *Structural and Multidisciplinary Optimization*.

[10] Chien Y.-H., Yao C.-K., Chao Y.-H. (2020). Effects of multidisciplinary participatory design method on students’ engineering design process. *Engineering Times*.

[11] Kontogiannis S. G., Savill M. A. (2020). A generalized methodology for multidisciplinary design optimization using surrogate modelling and multifidelity analysis,” Optimization and Engineering. *Optimization and Engineering*.

[12] Fredrik E., Tim H., Joel J. (2020). Multidisciplinary design automation – a conceptual framework for working with product model extensions. *International Journal of Agile Systems and Management*.

[13] Prestes Joly M., Teixeira J. G., Patrício L., Sangiorgi D. (2019). Leveraging service design as a multidisciplinary approach to service innovation. *Journal of Service Management*.

[14] Mengoni M., Germani M. (2009). Reverse Engineering and restyling of aesthetic products based on sketches interpretation. *Research in Engineering Design*.

[15] Milazzo M., Spezzaneve A., Persichetti A. (2020). Digital and experimental synergies to design high-heeled shoes. *International Journal of Advanced Manufacturing Technology*.

[16] Relvas C., Ramos A., Completo A., Simões J. A. (2011). Accuracy control of complex surfaces in reverse engineering. *International Journal of Precision Engineering and Manufacturing*.

[17] Fischer A., Park S. (1999). Reverse engineering: multilevel-of-detail models for design and manufacturing. *International Journal of Advanced Manufacturing Technology*.

[18] Yao A. W. L. (2005). Applications of 3D scanning and reverse engineering techniques for quality control of quick response products. *International Journal of Advanced Manufacturing Technology*.

[19] Curtis S. K., Harston S. P., Mattson C. A. (2011). The fundamentals of barriers to reverse engineering and their implementation into mechanical components. *Research in Engineering Design*.

[20] Iuliano L., Minetola P. (2009). Enhancing moulds manufacturing by means of reverse engineering. *International Journal of Advanced Manufacturing Technology*.

[21] Vijayan S., Xu Z., Zhou H. Application of intelligent systems in multi-modal information analytics.

